# Erosive Tooth Wear and Associated Risk Indicators in Schoolchildren from Rural and Urban Areas of the State of Mexico: A Cross-Sectional Study

**DOI:** 10.3390/children11091090

**Published:** 2024-09-06

**Authors:** José Cuauhtémoc Jiménez-Núñez, Álvaro Edgar González-Aragón Pineda, Teresa Villanueva-Gutiérrez, Rodrigo Leopoldo Longinos-Huerta, Luis Pablo Cruz-Hervert, Adrian Lussi, Socorro Aída Borges-Yáñez

**Affiliations:** 1 Master and Doctoral Program in Medical-Dental and Health Sciences at the School of Dentistry, National Autonomous University of Mexico, Ciudad de México 04510, Mexico; cuauhtemoc.jimenez@iztacala.unam.mx (J.C.J.-N.); rodrigo_longinos@comunidad.unam.mx (R.L.L.-H.); 2 Faculty of Higher Studies (FES) Iztacala, National Autonomous University of Mexico, Tlalnepantla de Baz 54090, Mexico; 3 Health Care Department, Metropolitan Autonomous University-Xochimilco, Ciudad de México 04960, Mexico; tvillanueva@correo.xoc.uam.mx; 4 Dental Public Health Department, Graduate and Research Division at the School of Dentistry, National Autonomous University of Mexico, Ciudad de México 04510, Mexico; lpcruzhervert@comunidad.unam.mx (L.P.C.-H.);; 5 University Hospital for Conservative Dentistry and Periodontology, Medical University of Innsbruck, 6020 Innsbruck, Austria; 6 School of Dental Medicine, University of Bern, 3010 Bern, Switzerland

**Keywords:** erosive tooth wear, dental erosion, prevalence, risk indicators, rural area, urban area

## Abstract

Background and Objective: Limited access to health services and low educational levels are factors in the rural population that are associated with the development of oral pathologies. However, the specific risk indicators contributing to erosive tooth wear (ETW) in these populations remain unclear. The objective of this study was to identify risk indicators associated with the prevalence of erosive tooth wear (ETW) in schoolchildren aged 8–12 years from rural and urban areas in the State of Mexico. Methods: A cross-sectional study was carried out in public schools in rural and urban areas. The prevalence of ETW was evaluated using the Basic Erosive Wear Examination (BEWE). The risk indicators studied were gastroesophageal reflux, vomiting, vitamin C, food, beverages, dental hygiene, bruxism, and hyposalivation. Logistic regression models were used to calculate odds ratios (OR) and 95% confidence intervals (CI). Results: The prevalence of ETW was higher in the rural area (77.3%) compared to the urban area (51.2%) (*p* = 0.001). The odds of presenting ETW were more than twice in schoolchildren from rural areas compared to those from urban areas (OR: 2.25, 95% CI: 1.11–3.98). Risk indicators varied between rural and urban populations, with different factors such as the consumption of fresh tomato sauce, orange, tangerine, atole (artisanal corn-based drink), and teeth grinding in the rural area, and lemon, soft drink, fruit juice consumption, and the simplified oral hygiene index in the urban area (*p* < 0.05). Conclusions: To prevent ETW, strategies offering tailored dietary and hygiene advice should be proposed, considering the specific conditions of each geographic area.

## 1. Introduction

Teeth endure various forms of physical and chemical damage throughout life, leading to wear [1]. Erosive tooth wear (ETW) is defined as a chemical–mechanical process that results in a cumulative loss of mineralized dental tissue not caused by bacteria [2]. Approximately 30% of children and adolescents suffer from ETW, and its prevalence is believed to be increasing [3]. Its etiology is multifactorial and is related to nutritional factors and factors related to the patient [4]. The main factor is the exposure of the teeth to acids, which can come from two sources: (a) intrinsic due to gastroesophageal reflux disease or frequent vomiting [1], and (b) extrinsic when related to the consumption of food, beverages, and drugs with a low pH [5,6].

In order to prevent the development of erosive tooth wear, which is a multifactorial disorder, it is crucial to identify patients at risk and evaluate potential risk factors. The risk factors that have been studied include sociodemographics, socioeconomics, general health, oral diseases, medication, oral hygiene, food, beverages, and dietary habits [7].

Previous studies have examined the connection between ETW and sociodemographic factors like geographic area [8,9,10,11,12,13,14]. The behavior and lifestyle of individuals can be influenced by whether they live in a rural or urban area, which can also expose them to varying risk factors for certain diseases [15]. Regardless of their health systems’ nature, scope, and efficiency, several countries have identified inequalities in access to dental services. The disparity in oral health based on social factors can be observed when marginalized individuals receive healthcare that does not adequately address their environmental needs [16,17]. Limited access to health services and low education levels in rural populations have been linked to the development of diseases like caries and periodontal diseases [18]. Research has shown that individuals in urban areas generally have better knowledge of dental hygiene compared to those in rural areas [19,20]. There is disagreement in the results concerning ETW. On one side, living in a rural area has been linked to a greater occurrence of ETW [10,14]. Meanwhile, others have reported a higher prevalence in urban areas [8,9]. Conversely, some studies have found no association [11,12,13].

In Mexico, the National Institute of Statistics and Geography (in Spanish, INEGI) reported that in 2020, 79% of the population lived in urban settlements, while only 21% settled in rural areas [21]. Industrialization, rapid urbanization, and globalization have significantly impacted eating habits in recent years, leading to a preference for manufactured and ready-to-eat foods [22]. The National Health and Nutrition Survey (in Spanish, ENSANUT) revealed that both rural and urban communities in Mexico showed patterns of consuming processed foods and beverages [23]. Thus, this study aims to identify risk indicators associated with the prevalence of erosive tooth wear (ETW) in schoolchildren aged 8–12 years from rural and urban areas in the State of Mexico.

## 2. Materials and Methods

A cross-sectional study was carried out in public schools in the municipalities of Acambay de Ruiz Castañeda and Tlalnepantla de Baz, state of Mexico. Sampling was made by convenience and was carried out between August and December 2023. The study was carried out under the guidelines established in the STROBE Initiative Declaration (Strengthening the Reporting of Observational studies in Epidemiology) [24].

### 2.1. Participants

The INEGI classifies localities as either rural or urban based on population density. San Antonio Detiña is a rural community in Acambay with 2230 residents who prioritize agriculture [21]. Around 44.3% of the population belongs to the Otomí ethnic group, making it an indigenous community as well. However, the community of San Bartolo Tenayuca in Tlalnepantla is primarily an urban area with approximately 20,000 inhabitants, with a strong emphasis on industry and commerce [25]. The average duration of schooling in Acambay is eight years, compared to 11 years in Tlanepantla. In Acambay, the illiteracy rate for people over 15 years old is 8.0%, compared to 1.8% in Tlanepantla [21].

To determine differences between two proportions, a sample size calculation was conducted using data from a prior study [11], with a 95% confidence level and a 10% margin of error. In order to account for a 15% non-response rate, 206 subjects were invited in each group (rural and urban areas). The study included children between the ages of 8 and 12 who attended schools in the mentioned places. Schoolchildren with health conditions or fixed orthodontic appliances that impeded dental evaluation were excluded from the study.

The parents or guardians who agreed to participate signed an informed consent form, and the students were asked for their assent. The research protocol was evaluated and approved by the Ethics Committee of the Faculty of Higher Education Iztacala (CE/FESI/042022/1504), and Ethics and Research Committee of the Faculty of Dentistry (CIE/0105/03/2023), of the National Autonomous University of Mexico.

### 2.2. Variables

The prevalence of ETW was measured as the dependent variable, using the criteria of the Basic Erosive Wear Examination (BEWE). The prevalence of ETW was determined if the participant had at least one affected tooth (BEWE > 0). To determine severity, the participant was classified according to the maximum code found for any tooth. BEWE scores from the sum of the highest value of each sextant [26]. The main independent variable was the geographic area (rural/urban), according to the classification of the Single Catalog of Codes of State, Municipal and Local Geostatistical Areas of the INEGI [21]. The collected risk indicators included sociodemographic factors: age (years), sex (male/female), and indigenism (no/yes); general health conditions: gastroesophageal reflux (no/yes), frequent vomiting (no/yes), and vitamin C consumption (no/yes); dietary habits: daily food and drink consumption (no/yes), keep beverages held in the mouth (no/yes), and consumption of acidic drinks before sleeping (no/yes); dental hygiene: simplified oral hygiene index [OHI-S] (score average) [27], tooth brushing frequency (once/twice of more), and topical fluoride application (no/yes); bruxism: grinding teeth at night (no/yes), temporomandibular joint pain (no/yes), clench teeth (no/yes), and grinding teeth during the day (no/yes); and hyposalivation (no/yes).

### 2.3. Data Collection

#### 2.3.1. Questionnaire

A questionnaire from previous studies [28,29] was used to collect risk indicator information. It was evaluated for this study and administered to 30 schoolchildren (not included in the study sample). An internal consistency of 0.87 (Cronbach’s alpha), a test–retest reliability of 0.89 (intraclass correlation coefficient), and an area under the Receiver Operating Characteristic (ROC) curve of 0.96 was obtained. Each child was interviewed by trained and standardized interviewers to ensure unbiased responses.

#### 2.3.2. Dental Evaluation

**Figure 1:**
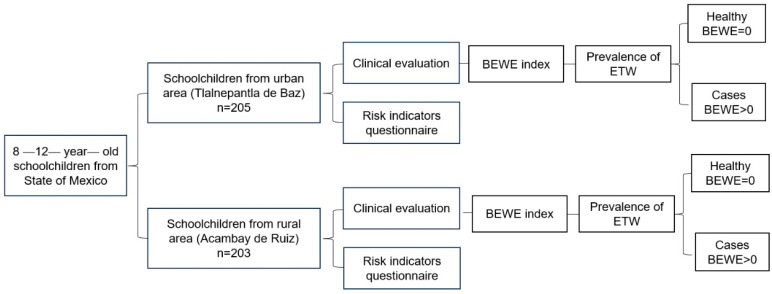
Data collection process.

Dental evaluation was performed by a standardized examiner on the BEWE and the OHI-S [27]. Standardization involved both theoretical and practical sessions that included study models, photographs, and participants who were the same age but not part of the study. Subsequently, inter-rater reliability against the expert and intra-rater reliability were evaluated in 30 participants, where kappa coefficients were obtained for both indices (BEWE = 0.886, and OHI-S = 0.846). Dental evaluations were performed in a classroom within each school. For the dental evaluation, the participant lay down on a portable table and artificial light was used to illuminate the oral cavity. First, oral hygiene was evaluated and then the presence of ETW. A PCP11 probe (Hu-Friedy, Chicago, IL, USA), a dental mirror (Arain, Sialkot, Punjab, Pakistan), and gauze pads were used for the examination. For the ETW, the examiner evaluated all teeth present from buccal, palatal/lingual, and occlusal/incisal, regardless of dentition type. The examiner identified ETW according to the following codes: 0 = when there was no evidence of ETW; 1 = initial enamel loss; 2 = distinctive defect less than 50% of the surface; and 3 = distinctive defect greater than 50% of the surface. In case of doubt between two criteria, the examiner chose the lower criterion. If the tooth had any extensive restoration (>1/3), the involved surface was excluded [29]. Hyposalivation was assessed using the wafer test [30], for which a wafer was placed on the back of the schoolchild’s tongue and the time until it diluted was counted (>4 min = hyposalivation). Figure 1 presents the diagram of the data collection process.

### 2.4. Analysis of Data

The data were recorded in the Epidata Entry 3.1 program (Epidata Association, Odense, Denmark) and were analyzed with the Stata 17 software (Stata Corp., College Station, TX, USA). Data were collected on the prevalence and regional distribution of ETW, along with the occurrence of risk factors. Then, a bivariate analysis was used to examine the relationships among ETW prevalence, geographic area, and risk indicators. For the bivariate analysis, the Chi-square test was used for categorical data and the Mann–Whitney U test for numeric data. A value of *p* < 0.05 was considered statistically significant. Two logistic regression models were conducted for multivariate analysis: one for the rural area, and one for the urban area. The criterion for including variables in the model was a *p* value ≤ 0.25. Odds ratios (OR) and 95% confidence intervals (CI) were calculated. A result was considered statistically significant if the 95% confidence interval did not include unity and a value of *p* < 0.05. Interactions were also evaluated.

## 3. Results

The study included 203 rural schoolchildren and 205 urban schoolchildren (*n* = 408), with a nonresponse rate of 1.0% (4/412). Women made up 52.7% with an average age of 10.00 ± 0.92 years. Schoolchildren had an average of 22.34 ± 2.63 total teeth, with 4.60 ± 4.07 deciduous teeth and 17.74 ± 5.16 permanent teeth.

### 3.1. Erosive Tooth Wear

**Figure 2:**
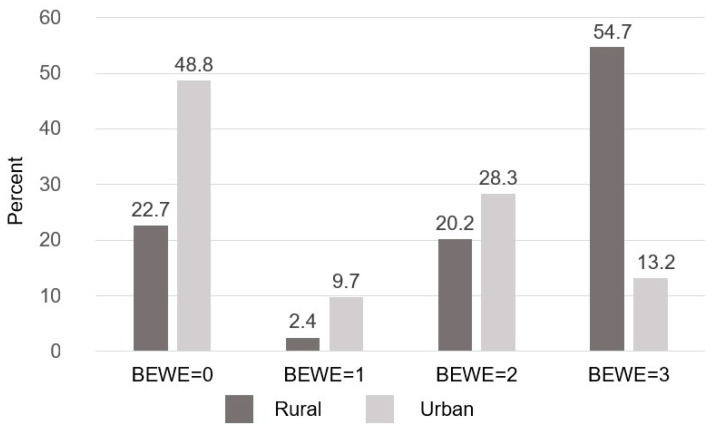
Distribution and severity of erosive dental wear based on schoolchildren’s geographic area in the State of Mexico.

ETW was found in 64.2% of cases (*n* = 262), with a higher prevalence in the rural area (77.3%) compared to the urban area (51.2%) (*p* < 0.001) (Table 1). The odds of presenting ETW increased by more than twice in schoolchildren from the rural area than from the urban area (OR: 2.25, 95% CI, 1.11–3.98). The prevalence of the most severe degree of ETW (BEWE = 3) was higher in the rural area (54.6%) compared to the urban area (13.17%) (*p* = 0.001). Likewise, there was a higher BEWE score in the rural area 6.76 ± 5.42, than in the urban area 2.24 ± 3.18 *(p* < 0.001) (Figure 2).

### 3.2. Risk Indicators

In both areas, schoolchildren with ETW were younger compared to those who did not have ETW (*p* < 0.05). There were no significant differences based on sex, indigenism, and general health variables (*p* > 0.05) (Table 2).

In the rural area, daily consumption of oranges, tangerines, pickled chilies, and fresh tomato sauce was associated with ETW (*p* < 0.05). In the urban area, foods associated with ETW included lemons and strawberries (*p* < 0.05) (Table 3).

In the urban area, the presence of ETW was linked to the daily consumption of soft drinks and fruit juice (*p* < 0.05). In the rural area, no differences were found according to daily beverage consumption (*p* > 0.05) (Table 4).

According to the OHI-S, in the urban area, those with ETW had a higher score (1.20 ± 0.61) compared to those without ETW (1.03 ± 0.47) (*p* = 0.032). No differences were found regarding bruxism variables (Table 5).

### 3.3. Multivariate Analysis

The logistic regression model revealed that being a member of the rural population with daily consumption of fresh tomato sauce increased the odds of presenting ETW three times (OR: 3.00, 95% CI, 1.29–6.98, *p* = 0.010), more than three times for daily orange consumption (OR: 3.86, 95% CI, 1.42–10.48, *p* = 0.008) and mandarin (OR: 3.24, 95% CI, 1.25–8.41, *p* = 0.016). Daily consumption of atole (an artisanal corn-based drink) decreased the odds of presenting ETW by 70% (OR: 0.30, 95% CI 0.12–0.73, *p* = 0.008), while grinding teeth during the day also increased the odds of presenting ETW by three times (OR: 3.15, 95% CI 1.01–9.84, *p* = 0.048).

The urban population with daily lemon consumption increased the odds of presenting ETW by almost three times (OR: 2.99, 95% CI 1.57–5.71, *p* = 0.001). Daily consumption of soft drinks increased the odds of presenting ETW more than twice (OR: 2.42, 95% CI 1.25–4.67, *p* = 0.008), and daily consumption of fruit juices more than three times (OR: 3.62, 95% CI 1.05–12.52, *p* = 0.041). Finally, it was found that the increase in the OHI-S score increased the odds of presenting ETW by 90% (OR:1.90 95% CI, 1.03–3.48, *p* = 0.038).

Table 6 presents the raw and adjusted odds ratios for the rural area, and for the urban area.

## 4. Discussion

This pioneering study reveals significant geographical variations in erosive tooth wear (ETW) in Mexico, with a higher prevalence in rural areas (77.3%) compared to urban areas (51.2%). Using the BEWE index, it identifies distinct risk indicators for each setting, underscoring the need for tailored prevention strategies. According to this study, the occurrence of ETW in the school population varied depending on the geographical area. Similarly, it enabled us to analyze both the overall and specific risk indicators for every area. The main strength of this work is that it is the first to evaluate ETW in rural areas carried out in Mexico and the second in Latin America [11]. By utilizing the BEWE index, which has been extensively employed in recent years, valuable information was obtained about the connection between ETW and sociodemographic factors like geographical area. Regarding the study’s limitations, it should be noted that self-reporting was used to collect information like gastroesophageal reflux, frequent vomiting, and bruxism, instead of more specialized methods. Another constraint is the inability to establish causality due to the cross-sectional nature of the study.

The prevalence of ETW varies depending on the geographical area, with studies suggesting a higher rate in rural areas compared to urban areas. These have been reported from 40.0% in adults in Nigeria a [12] and Europe [10], and 66.5% in Nepalese children and adolescents aged 5 to 15 years using the BEWE index [14]. The results of this study had similar findings to the previously mentioned studies since the prevalence of ETW in the rural area was higher than in the urban area (77.3% vs. 51.2%).

The explanation for these findings could be that disadvantaged populations in Mexico, particularly those in rural areas [31], are more likely to have poor oral health due to the social gradient in health [32]. Among the factors that explain this gradient, political and infrastructure issues can be considered, where healthcare services in rural areas are usually very basic and lack adequately equipped hospitals or health clinics [33]. The study shows that just 17.5% of the rural population studied has access to a healthcare-providing social security institution, in contrast to the 85.1% of the urban population who are insured [21].

Another related factor that could explain this is education. Rural areas in Mexico generally have lower levels of educational achievement [34]. This study examines the educational differences between rural and urban populations, with rural schoolchildren having lower average years of schooling (8 vs. 11) and a higher illiteracy rate (8.0% vs. 1.8%) [21].

Two studies conducted in Hungary [9], and India [8], reported a higher prevalence of ETW in urban areas compared to rural areas. The prevalence of ETW measured by the BEWE index was 44.3%, 49.0%, and 45.0% in these countries, respectively. In this study, the prevalence of this condition in the urban area was 51.2%. Evidence suggests that there is a heightened likelihood of ETW occurrence in urban populations of children and adolescents in Mexico [28,29].

The likelihood of having ETW in the rural area increased with the higher daily consumption of oranges (OR = 3.86) and tangerines (OR = 3.24). Other studies have also found that daily intake of citrus fruits is crucial in the development of ETW [35,36]. Similarly, consuming fresh tomato sauce daily increased the chances of developing ETW (OR = 3.00). Tomato is an acidic fruit with a pH of 4.6 [37], which has the capacity to dissolve hard tooth tissues [38]. Finally, schoolchildren from rural areas who reported grinding their teeth had a higher chance of presenting ETW (OR = 3.15), which has already been reported in previous studies [4].

On the other hand, in the rural area, daily consumption of atole (an artisanal corn-based drink) was found to have a protective effect (OR = 0.30). The preference for this drink over processed options like juices and soft drinks may be influenced by cultural factors. Besides, corn atole contains a significant amount of calcium hydroxide, making it highly alkaline [39]. Products high in calcium, such as milk and natural yogurt, have been reported to be protective for ETW [5,40].

In the urban area, daily consumption of lemon (OR = 2.99) and fruit juices (OR = 3.62) increased the odds of developing ETW. These associated factors are also consistent with what was reported in previous studies [28,41]. Soft drinks increased the odds of presenting ETW (OR = 2.42). This was an expected factor, given that it is consistently reported among studies [10,35]. Although the association was established with daily intake of this acidic beverage, no association was found with drinking behaviors like retaining it in the mouth or consuming it prior to sleep. However, studies have shown that the pH of the tooth surface and the risk of ETW are strongly influenced by the drinking method [42].

Similarly, in the urban area, the odds of presenting ETW were higher in cases of poor hygiene (OR = 1.90). This association may be related to insufficient hygiene habits that might restrict access to protective factors, such as fluoride, which are present in toothpastes and mouthwashes [43].

Patients who show a risk of erosive dental wear should be advised to undergo preventive care early on, in order to avoid its occurrence and advancement over time [44]. The findings of the current study suggest that future research should prioritize the development of interventions targeting the risk indicators identified, with the goal of reducing the prevalence of ETW in school-age children. Understanding and addressing the barriers that children may encounter is essential in rural areas to prevent the development and progression of such injuries.

## 5. Conclusions

The prevalence of ETW was higher in the rural area (77.3%) compared to the urban area (51.2%). These contrasting prevalence rates can be attributed to variations in dietary habits, such as the consumption of citrus fruits, soft drinks, fruit juices, and corn atole. To prevent ETW, it is important to propose strategies that offer tailored dietary and hygienic advice to schoolchildren and their caregivers, considering the conditions of each geographical area.

## Figures and Tables

**Table 1:** Erosive tooth wear (ETW) prevalence, severity and score based on schoolchildren’s geographic area in the State of Mexico.

		Total (%) *n* = 408 (100.0)	Rural (%) *n* = 203 (100.0)	Urban (%) *n* = 205 (100.0)	*p*
Prevalence ETW	BEWE = 0	146 (35.8)	46 (22.7)	100 (48.8)	0.001 *
	BEWE > 0	262 (64.2)	157 (77.3)	105 (51.2)	
Severity ETW	BEWE = 0	146 (35.8)	46 (22.7)	100 (48.8)	0.001 *
	BEWE = 1	25 (6.1)	5 (2.4)	20 (9.7)	
	BEWE = 2	99 (24.3)	41 (20.2)	58 (28.3)	
	BEWE = 3	138 (33.8)	111 (54.7)	27 (13.2)	
Average BEWE Score ± s.d.		4.48 ± 4.97	6.76 ± 5.42	2.24 ± 3.18	0.001 **

* Chi-square test, ** Mann–Whitney U test, BEWE = Basic Erosive Wear Examination, s.d. = standard deviation.

**Table 2:** Prevalence of erosive tooth wear (ETW) according to sociodemographic variables and general health based on schoolchildren’s geographic area in the State of Mexico.

		Rural (%) *n* = 203 (100.0)			Urban (%) *n* = 205 (100.0)		
		BEWE = 0 (%) *n* = 46 (22.7)	BEWE > 0 (%) *n* = 157 (77.3)	*p* *	BEWE = 0 (%) *n* = 100 (48.8)	BEWE > 0 (%) *n* = 105 (51.2)	*p* *
Average age ± s.d.		10.21 ± 0.89	9.83 ± 0.89	0.013 **	10.29 ± 0.85	9.89 ± 0.99	0.002 **
Sex	Male	20 (19.4)	83 (80.6)		39 (43.3)	51 (56.7)	
	Female	26 (26.0)	74 (74.0)	0.263	61 (53.0)	54 (47.0)	0.167
Indigenous	No	8 (24.2)	25 (75.8)		90 (47.9)	98 (52.1)	
	Yes	38 (22.3)	132 (77.7)	0.812	10 (58.8)	7 (41.2)	0.387
Gastroesophageal reflux	No	41 (22.6)	140 (77.4)		90 (47.6)	99 (52.4)	
	Yes	5 (22.7)	17 (77.3)	0.994	10 (62.5)	6 (37.5)	0.253
Frequent vomiting	No	39 (22.2)	137 (77.8)		91 (49.5)	93 (50.5)	
	Yes	7 (25.9)	20 (74.1)	0.663	9 (42.9)	12(57.1)	0.567
Vitamin C	No	34 (23.6)	110 (76.4)		68 (50.0)	68 (50.0)	
	Yes	12 (20.3)	47 (79.7)	0.613	32 (46.4)	37 (53.6)	0.624

* Chi-square test, ** Mann–Whitney U test, BEWE = Basic Erosive Wear Examination, s.d. = standard deviation.

**Table 3:** Prevalence of erosive tooth wear (ETW) according to the daily food consumption based on schoolchildren’s geographic area in the State of Mexico.

		Rural (%) *n* = 203 (100.0)			Urban (%) *n* = 205 (100.0)		
Daily Consumption		BEWE = 0 (%) *n* = 46 (22.7)	BEWE > 0 (%) *n* = 157 (77.3)	*p* *	BEWE = 0 (%) *n* = 100 (48.8)	BEWE > 0 (%) *n* = 105 (51.2)	*p* *
Apple	No	33 (23.7)	106 (76.3)	0.588	77 (49.0)	80 (51.0)	
	Yes	13 (20.3)	51 (79.7)		23 (47.9)	25 (52.1)	0.891
Lemon	No	34 (25.8)	98 (74.2)	0.151	59 (62.1)	36 (37.9)	
	Yes	12 (16.9)	59 (83.1)		41 (37.3)	69 (62.7)	0.001
Orange	No	37 (31.9)	79 (68.1)	0.001	76 (51.7)	71 (48.3)	
	Yes	9 (10.3)	78 (89.7)		24 (41.4)	34 (58.6)	0.183
Tangerine	No	36 (31.9)	77 (68.1)		59 (53.6)	51 (46.4)	
	Yes	10 (11.1)	80 (88.9)	0.001	41 (43.2)	64 (56.8)	0.134
Strawberries	No	29 (24.4)	90 (75.6)		79 (54.9)	65 (45.1)	
	Yes	17 (20.2)	67 (79.8)	0.489	21 (34.4)	40 (65.6)	0.007
Grapes	No	30 (23.4)	98 (76.6)		62 (48.8)	65 (51.2)	
	Yes	16 (21.3)	59 (78.7)	0.730	38 (48.7)	40 (51.3)	0.989
Peach	No	39 (24.1)	123 (75.9)		92 (48.9)	96 (51.1)	
	Yes	7 (17.1)	34 (82.9)	0.339	8 (47.1)	9 (52.9)	0.882
Cheese	No	36 (22.1)	127 (77.9)		77 (45.8)	91 (54.2)	
	Yes	10 (25.0)	44 (75.0)	0.693	23 (62.2)	14 (37.8)	0.072
Hot sauce	No	40 (25.3)	118 (74.7)		64 (46.7)	73 (53.3)	
	Yes	6 (13.3)	39 (86.7)	0.090	36 (52.9)	32 (47.1)	0.401
Pickled chili peppers	No	44 (29.3)	104 (70.3)		93 (49.7)	94 (50.3)	
	Yes	2 (3.6)	53 (96.4)	0.001 **	7 (38.9)	11 (61.1)	0.379
Ketchup	No	37 (20.9)	140 (79.1)		88 (49.2)	91 (50.8)	
	Yes	9 (34.6)	17 (65.4)	0.119	12 (46.1)	14 (53.9)	0.774
Fresh tomato sauce	No	34 (29.1)	83 (70.9)		81 (48.5)	86 (51.5)	
	Yes	12 (13.9)	74 86.1)	0.011	19 (50.0)	19 (50.0)	0.868
Dressing	No	44 (22.8)	149 (77.2)		96 (49.2)	99 (50.8)	
	Yes	2 (20.0)	8 (80.0)	0.837 **	4 (40.0)	6 (60.0)	0.569 **
Vinegar	No	44 (22.0)	156 (78.0)		100 (49.0)	104 (51.0)	
	Yes	2 (66.7)	1 (33.3)	0.067 **	0 (0.0)	2 (100.0)	0.328 **

* Chi-square test, ** Fisher’s exact test, BEWE = Basic Erosive Wear Examination.

**Table 4:** Prevalence of erosive tooth wear (ETW) according to the daily beverage consumption based on schoolchildren’s geographic area in the State of Mexico.

		Rural (%) *n* = 203 (100.0)			Urban (%) *n* = 205 (100.0)		
Daily Consumption		BEWE = 0 (%) *n* = 46 (22.7)	BEWE > 0 (%) *n* = 157 (77.3)	*p* *	BEWE = 0 (%) *n* = 100 (48.8)	BEWE > 0 (%) *n* = 105 (51.2)	*p* *
Fruit yogurt	No	34 (23.1)	113 (76.9)		83 (51.9)	77 (48.1)	
	Yes	12 (21.4)	44 (78.6)	0.796	17 (37.8)	28 (62.2)	0.095
Milk	No	32 (26.0)	91 (74.0)		48 (46.1)	56 (53.9)	
	Yes	14 (17.5)	66 (82.5)	0.157	52 (51.5)	49 (48.5)	0.445
Soft drinks	No	32 (21.3)	118 (78.7)		74 (58.3)	53 (41.7)	
	Yes	14 (26.4)	39 (73.6)	0.447	26 (33.3)	52 (66.7)	0.001
Sport drinks	No	0 (0.0)	4 (100.0)		81 (50.3)	80 (49.7)	
	Yes	46 (23.1)	153 (76.9)	0.576 **	19 (43.2)	25 (56.8)	0.496
Energy drinks	No	42 (22.3)	146 (77.7)		94 (49.7)	95 (50.3)	
	Yes	4 (26.7)	11 (73.3)	0.700 **	6 (37.5)	10 (62.5)	0.347
Coffee	No	38 (23.5)	124 (76.5)		76 (48.4)	81 (51.6)	
	Yes	8 (19.5)	33 (80.5)	0.590	24 (50.0)	24 (50.0)	0.847
Tea	No	14 (29.2)	34 (70.8)		77 (47.8)	84 (52.2)	
	Yes	32 (20.7)	123 (79.3)	0.218	23 (52.3)	21 (47.7)	0.601
Fruit juice	No	40 (24.8)	121 (75.2)		96 (52.2)	88 (47.8)	0.004 **
	Yes	6 (14.3)	36 (85.7)	0.145	4 (19.0)	17 (81.0)	
Atole ***	No	31 (20.5)	120 (79.5)		91 (50.8)	88 (49.2)	
	Yes	15 (28.8)	37 (71.2)	0.217	9 (34.6)	17 (65.4)	0.122
Keep beverages held in the mouth	No	38 (22.3)	132 (77.7)		77 (48.4)	82 (51.6)	
	Yes	9 (26.7)	24 (73.3)	0.605	23 (50.0)	23 (50.0)	0.851
Consumption of acidic drinks before sleeping	No	42 (23.5)	137 (76.5)		91 (47.9)	99 (52.1)	
	Yes	4 (16.7)	20 (83.3)	0.455 **	9 (60.0)	6 (40.0)	0.367

* Chi-square test, ** Fisher’s exact test, *** Artisanal corn-based drink, BEWE = Basic Erosive Wear Examination.

**Table 5:** Prevalence of erosive tooth wear (ETW) according to dental hygiene, bruxism and hyposalivation based on schoolchildren’s geographic area in the State of Mexico.

		Rural (%) *n* = 203 (100.0)			Urban (%) *n* = 205 (100.0)		
		BEWE = 0 (%) *n* = 46 (22.7)	BEWE > 0 (%) *n* = 157 (77.3)	*p* *	BEWE = 0 (%) *n* = 100 (48.8)	BEWE > 0 (%) *n* = 105 (51.2)	*p* *
Average OHI-S ± s.d.		1.72 ± 0.66	1.79 ± 0.61	0.475 **	1.03 ± 0.47	1.20 ± 0.61	0.032 **
Tooth brushing frequency	1 time	12 (21.0)	45 (79.0)		13 (52.0)	12 (48.0)	
	2 times or more	34 (23.3)	112 (76.7)	0.732	87 (48.3)	93 (51.7)	0.731
Topical fluoride	No	32 (23.4)	105 (76.6)		75 (47.5)	83 (52.5)	
	Yes	14 (21.2)	52 (78.8)	0.732	25 (53.2)	22 (46.8)	0.491
Hyposalivation	No	43 (23.1)	143 (76.9)		98 (49.2)	101 (50.8)	
	Yes	3 (17.6)	14 (82.4)	0.606 ***	2 (33.3)	4 (66.7)	0.442 ***
Grinding teeth at night	No	39 (23.3)	128 (76.7)		93 (49.7)	94 (50.3)	
	Yes	7 (19.4)	29 (80.6)	0.611	7 (38.9)	11 (61.1)	0.379
Temporomandibular joint pain	No	35 (21.7)	126 (78.3)		80 (51.0)	77 (49.0)	
	Yes	11 (26.2)	31 (73.8)	0.539	20 (41.7)	28 (58.3)	0.260
Clench teeth	No	30 (22.2)	105 (77.8)		75 (47.5)	83 (52.5)	
	Yes	16 (23.5)	52 (76.5)	0.834	25 (53.2)	22 (46.8)	0.491
Grinding teeth during the day	No	41 (24.8)	124 (75.2)		89 (51.1)	85 (48.9)	
	Yes	5 (13.2)	33 (86.8)	0.121	11 (35.5)	20 (64.5)	0.108

* Chi-square test, ** Mann–Whitney U test, *** Fisher’s exact test, OHI-S = simplified oral hygiene index, BEWE = Basic Erosive Wear Examination, s.d. = standard deviation.

**Table 6:** Odds ratios (OR) and confidence intervals (CI) of the logistic regression models for prevalence of erosive dental wear according to each geographical area and risk indicators.

Variables	Rural Area Model *n* = 203		Urban Area Model *n* = 205	
	Crude OR (CI 95%)	Adjusted OR (CI 95%) **	Crude OR (CI 95%)	Adjusted OR(CI 95%) **
Fresh tomato sauce	2.75 (1.21–5.23) *	3.00 (1.29–6.98) *	-	-
Orange	4.05 (1.83–8.96) *	3.86 (1.42–10.48) *	-	-
Tangerine	3.54 (1.73–8.05) *	3.24 (1.25–8.41) *	-	-
Lemon	-	-	2.75 (1.56–4.86) *	2.99 (1.57–5.71) *
Soft drinks	-	-	2.49 (1.55–5.02) *	2.42 (1.25–4.67) *
Fruit juice	-	-	3.63 (1.50–14.30) *	3.62 (1.05–12.52) *
Atole ***	0.32 (0.31–1.10)	0.30 (0.12–0.73) *	-	-
Grinding teeth during the day	2.92 (0.79–5.95)	3.15 (1.01–9.84) *	2.02 (0.91–4.44)	1.99 (0.82–4.80)
OHI-S	1.31 (0.71–2.06)	1.38 (0.73–2.62)	1.82 (1.07–3.07) *	1.90 (1.03–3.48) *

* *p* < 0.05, ** adjusted for age and sex, *** artisanal corn-based drink, OHI-S = simplified oral hygiene index. References: daily consumption of food and drinks = no; grinding teeth = no; OHI-S < 1 point; hyposalivation = no.

## Data Availability

The original contributions presented in the study are included in the article. Further inquiries can be directed to the corresponding author.
